# Comorbidity of migraine and fibromyalgia in patients with cluster headache: psychological burden and healthcare resource utilization. A cross-sectional study

**DOI:** 10.22514/jofph.2024.004

**Published:** 2024-03-12

**Authors:** Elena P. Calandre, Jorge L. Ordoñez-Carrasco, Mahmoud Slim, Fernando Rico-Villademoros, Juan M. Garcia-Leiva

**Affiliations:** ^1^Institute of neurosciences, University of Granada, 18016 Granada, Spain; ^2^Department of Psychology and Sociology, University of Zaragoza (Campus Teruel), 4403 Teruel, Spain; ^3^Evidence Synthesis, Modelling & Simulation, Evidera, St. Laurent, QC H47 1V6, Canada

**Keywords:** Cluster headache, Fibromyalgia, Migraine, Psychosocial burden, Health care resource utilization

## Abstract

The aim was to describe the comorbidity and impact of fibromyalgia and/or migraine on patients with cluster headache. Comorbid diseases can exacerbate the physical and psychological burden experienced by patients. The comorbidities of cluster headache have been scarcely investigated, with the exception of migraine, which is well-known to coexist with cluster headache. In contrast, the comorbidity of migraine and fibromyalgia has been well investigated and firmly established. An internet survey was uploaded to the webpage of a cluster headache patient association. The survey collected sociodemographic and clinical data, and patients completed questionnaires that assessed depression, sleep quality, health-related quality of life, and health care resource utilization (HCRU) over the preceding six months. Differences in total depression, sleep quality, and health-related quality of life scores among the groups were analyzed with the Kruskal-Wallis test, and differences in HCRU were analyzed with the chi-square test. Ninety-one patients with cluster headache participated in the survey; 39 (42.9%) experienced only cluster headache, 15 (16.5%) experienced cluster headache and migraine, 10 (11%) experienced cluster headache and fibromyalgia, and 27 (29.7%) experienced cluster headache with comorbid fibromyalgia and migraine. Moderate depression scores and positive suicidal ideation were found across all subgroups. Sleep quality and health-related quality of life were consistently poor across the different subgroups, with the cluster headache with comorbid fibromyalgia and migraine subgroup showing significantly lower scores. Heavy use of health care resources was observed across all subgroups, with no notable differences among them. The comorbidity of cluster headache with fibromyalgia and/or migraine does not seem to be infrequent. This comorbidity substantially increases the psychosocial burden experienced by patients and decreases their overall quality of life.

## 1. Introduction

Cluster headache, although the most common type of autonomic trigeminal cephalalgia, has a very low frequency of presentation, with a lifetime prevalence of approximately 124 per 100,000 [1]. Cluster headache pain is strictly unilateral, orbital, supraorbital and/or temporal, lasts for 15–180 minutes, and is associated with autonomic features such as lacrimation, nasal congestion, eyelid ptosis and miosis, among others. Episodic cluster attacks occur in series that last for weeks or months followed by long periods of remission, while chronic cluster headaches have few or no periods of remission [2]. Migraine is the second most common type of primary headache after tension-type headache and is one of the most disabling headache disorders [3]. Migraine pain can be unilateral or bilateral and is typically associated with photo- and/or phonophobia and nausea and/or vomiting [2]. Patients with cluster headache may present with some of the same features as migraine patients. Both cluster headache and migraine may coexist in the same patient, with an estimated prevalence of comorbidity ranging between 10% and 16.7% [4].

On the other hand, the comorbidity of migraine and fibromyalgia, both of which are included in the spectrum of central sensitization syndromes, has been extensively studied and shown to be bidirectional [5]. Fibromyalgia is a syndrome characterized by generalized pain of musculoskeletal characteristics associated, in most patients, with other symptoms such as sleep disturbances, cognitive impairment, chronic fatigue, depression, anxiety and gastrointestinal symptoms [6]. Among the scarce publications that have specifically investigated cluster headache comorbidities, only two studies have reported its comorbidity with fibromyalgia. The first study reported that 5 of the 75 (6.7%) patients with definite cluster headache were also diagnosed with fibromyalgia [7]. The second study found that 12 of the 499 (3%) patients with cluster headache were also diagnosed with fibromyalgia, with a higher prevalence of fibromyalgia in women than in men (6.9% *vs.* 1.1%) [8].

Cluster headache, migraine and fibromyalgia have been associated with depression [9, 10, 11], suicidal ideation [12, 13, 14], anxiety [10, 15, 16], and sleep problems [17, 18, 19]. These disorders have also been shown to be associated with an augmented use of health care resources and increased economic costs [20, 21, 22]. However, with the exception of comorbid migraine and fibromyalgia [23], there is no available evidence of the burden associated with the co-occurrence of these diseases.

Therefore, our study aimed to examine the comorbidity of migraine and/or fibromyalgia in a cohort of patients experiencing cluster headache. Specifically, we aimed to compare the psychosocial profiles, quality of life, and health care resource utilization (HCRU) of patients with noncomorbid cluster headache versus those with cluster headache associated with migraine and/or fibromyalgia.

## 2. Patients and methods

### 2.1 Design

This was an observational, cross-sectional Spanish study performed between 2020 and 2021 that included patients experiencing cluster headache. Patients were required to be aged 18 years or older and to have been diagnosed with cluster headache by a physician, regardless of whether they had any comorbidities. No additional inclusion criteria were required, and no specific exclusion criteria were established.

An online survey was uploaded to the patient association website “Asociación de cefalea en racimos y primarias de España” (Spanish association of cluster headache and other primary headaches). This association has members both in Spain and Spanish-speaking American countries, which enabled us to enroll more patients with cluster headaches. The members of the association were kindly asked to participate by completing the questionnaire online. The survey included information regarding the purpose of the study and that its completion required participants’ consent to participate. Patients were also informed that their participation was free and that they would not receive any kind of compensation. Sociodemographic data, including age, sex, marital status and educational status, were collected. Information related to additional physician-diagnosed comorbid diseases was also collected. The survey included a total of 60 items that could be completed in an average of 20 minutes.

### 2.2 Outcome measures

#### 2.2.1 Patients health questionnaire-9 (PHQ-9)

This 9-item questionnaire evaluates depressive symptomatology over the last two weeks. The total score ranges from 0 to 27, with higher scores indicating more severe depression. A cutoff point of 10 is used to identify patients with clinically relevant depression [24]. Scores of 1 to 4 are considered indicative of no depression, scores of 5 to 6 are considered indicative of mild depression, scores of 10 to 14 are considered indicative of moderate depression, scores of 15 to 19 are considered indicative of moderate to severe depression, and scores of 20 to 27 are considered indicative of severe depression. A positive response to the 9th or i item (“Thoughts that you would be better off dead or hurting yourself in some way?”) is considered an indicator of suicidal ideation; the severity of suicidal ideation is scored as “0” when the answer is “not at all”, “1” when the answer is “several days”, “2” when the answer is “more than half the days”, and “3” when the answer is “nearly every day”. Positive suicidal ideation was considered when the 9th item score was ≥1. The Spanish-validated version of the PHQ-9 was used [25]. The reliability estimates of the scores for this study using the Cronbach alpha coefficients were 0.88 for the fibromyalgia sample, 0.86 for the migraine sample, and 0.87 for the fibromyalgia plus comorbid migraine sample.

#### 2.2.2 Insomnia severity index (ISI)

This tool assesses individuals’ self-perceptions of insomnia symptoms as well as the distress caused by sleeping problems. The total score ranges from 0 to 28, with higher values indicating more relevant sleeping problems. A cutoff point of 10 is used to identify insomnia cases [26]. The Spanish-validated version of this questionnaire was used [27]. The reliability estimates of the scores for this study using the Cronbach alpha coefficients were 0.86 for the fibromyalgia sample, 0.88 for the migraine sample, and 0.85 for the fibromyalgia plus comorbid migraine sample.

#### 2.2.3 EuroQOL-5D-5L

The 5-level EQ-5D version (EQ-5D-5L) was introduced by the EuroQol Group in 2009. The questionnaire comprises five dimensions: mobility, self-care, usual activities, pain/discomfort and anxiety/depression. Each dimension has 5 levels: no problems, slight problems, moderate problems, severe problems and extreme problems. Scores range from −0.564 to 1.000, with higher scores indicating better health-related quality of life. The EQ-5D-5L also includes an EQ visual analog scale (VAS) that records a patient’s self-rated health on a vertical VAS, where the two extremes are labeled “The best health you can imagine” (100) and “The worst health you can imagine” (0). The EQ-5D-5L is available in 150 languages (EuroQol Research Foundation. EQ-5D-5L user guide, 2019. EuroQol Research Foundation) [28]. The mean values for the Spanish population are 0.897 for the EQ-5D-5L index score and 75.7 for the EQ-5D-5L VAS score [29]. The reliability estimates of the scores for this study using the Cronbach alpha coefficients were 0.70 for the fibromyalgia sample, 0.65 for the migraine sample, and 0.75 for the fibromyalgia plus comorbid migraine sample.

#### 2.2.4 Health care resources

An *ad hoc* questionnaire HCRU during the past six months was developed. The questionnaire captured the number of the following activities or events during the past six months: visits to a family doctor, visits to specialists, emergency room visits, medical analyses, hospitalization, and surgical interventions. The questionnaire was intended to cover the most frequent medical and surgical procedures the patients could have been exposed to during this time period. The specific reasons for surgical interventions and hospitalizations were queried using open-ended questions. The total number of each type of procedure was compared within groups.

### 2.3 Statistical analysis

Continuous data are presented as the means and standard deviations and were analyzed using the Kruskal-Wallis test and *post hoc* Dunn test for multiple comparisons. All continuous data, *i.e.*, age, PHQ-9 total score, ISI total score, and EQ-5D-5L total score were normally distributed according to the D’Agostino and Pearson test. Categorical data are presented as the absolute and relative frequencies and were analyzed with the Chi-square test. A *p* value < 0.05 was considered to indicate statistical significance. The statistical analysis was performed using GraphPad Prism, version 9.5.1 (GraphPad Software, San Diego, CA, USA).

## 3. Results

A total of 91 patients diagnosed with cluster headache completed the survey. Thirty-nine patients (42.9%) experienced cluster headache without comorbid migraine or fibromyalgia, 15 (16.5%) experienced cluster headache with comorbid migraine, 10 (11%) experienced cluster headache with comorbid fibromyalgia, and 27 (29.7%) experienced cluster headache associated with migraine and fibromyalgia. Sixty-eight participants lived in Spain, and the remaining 24 lived in Spanish-speaking American countries.

Table 1 shows the sociodemographic and clinical data of the total sample and the different subgroups. Male sex predominated among patients who experienced noncomorbid cluster headache (61.5% of the sample), whereas female sex predominated in those with comorbid migraine/fibromyalgia (73.3% in patients with cluster headache and migraine, 100% in patients with cluster headache and fibromyalgia, and 85.2% in patients with cluster headache, migraine and fibromyalgia). Most patients (62.3%) lived with a partner. The percentage of patients with a university education was the lowest among those with cluster headache associated with both migraine and fibromyalgia (*p *< 0.0001). The number of comorbid medical diseases was highest in patients with comorbid fibromyalgia (mean value: 2.01 ± 1.8) and lowest in patients with noncomorbid cluster headache (0.31 ± 1.0). Osteoarticular conditions (mainly osteoarthritis) were the most frequently associated medical diseases and were mostly observed in patients with cluster headache associated with fibromyalgia with or without comorbid migraine. Other common comorbidities included those related to mental health (mostly anxiety and/or depression) and gastrointestinal and cardiovascular disorders. Miscellaneous conditions such as chronic fatigue syndrome, multiple chemical sensitivity, and food intolerance were also especially frequent in patients with cluster headache and comorbid fibromyalgia with or without migraine.

**Table 1.** Sociodemographic and clinical data of the sample.

		Total sample (N = 91)	CH (N = 39)	CH + M (N = 15)	CH + FM (N = 10)	CH + M + FM (N = 27)	*p*
Female sex (N (%))		59 (65.8)	15 (38.5)	11 (73.3)	10 (100.0)	23 (85.2)	<0.0001
Age (mean ± SD)		44.8 ± 10.8	43.4 ± 11.5	43.1 ± 11.6	46.6 ± 10.7	47.2 ± 9.2	0.393
With partner (N (%))		57 (62.3)	21 (53.8)	10 (66.7)	7 (70.0)	19 (70.4)	0.509
University studies (N (%))		39 (42.8)	18 (46.2)	8 (53.3)	5 (50.0)	8 (29.6)	0.394
Employed (N (%))		40 (43.9)	17 (43.6)	9 (60.0)	7 (70.0)	18 (66.7)	0.997
# of comorbid medical diseases (range and mean ± SD)		(0–10) 1.23 ± 1.9	(0–6) 0.31 ± 1.0	(0–2) 0.54 ± 0.7	(0–5) 2.0 ± 1.8	(0–10) 2.67 ± 2.3*	<0.0001
Type of comorbid medical diseases (N (%))							
	Osteoarticular	30 (33.0)	2 (5.1)	0	6 (60.0)	22 (81.5)	
	Mental health	13 (14.3)	2 (5.1)	1 (6.7)	3 (30.0)	7 (25.9)	
	Gastrointestinal	12 (13.2)	2 (5.1)	0	4 (40.0)	6 (22.2)	
	Cardiovascular	10 (11.0)	2 (5.1)	0	2 (20.0)	6 (22.2)	
	Respiratory	5 (5.4)	1 (2.5)	1 (6.7)	0	3 (11.1)	
	Neurological	5 (5.4)	1 (2.5)	1 (6.7)	1 (10.0)	2 (7.4)	
	Renal	2 (2.2)	0	0	0	2 (7.4)	
	Metabolic	3 (3.3)	0	1 (6.7)	1 (10.0)	1 (3.7)	
	Hormonal	4 (4.4)	1 (2.5)	0	1 (10.0)	2 (7.4)	
	Others	26 (28.6)	2 (5.1)	1 (6.7)	10 (66.7)	13 (48.1)	

CH: cluster headache; FM: fibromyalgia; M: migraine; SD: standard deviation. *Significantly different from CH, M and CH + M; #: number. Statistical differences were evaluated across subgroups.

Table 2 summarizes the data related to the psychosocial profiles and quality of life results of the different subgroups. Overall, except for suicidal ideation, participants with the three comorbid conditions had poorer scores on the various psychosocial measures, but the differences across the subgroups were statistically significant only for sleep (*p* = 0.0048) and quality of life (*p *< 0.0001). Of the total sample, sixty (73.6%) patients had depression scores above the cutoff point; this was observed across all sample subgroups, with 27 patients (69.2%) having noncomorbid cluster headache, 10 patients (66.7%) having cluster headache and comorbid migraine, 7 patients (70%) having cluster headache and comorbid fibromyalgia, and 23 patients (85.2%) having cluster headache and comorbid migraine and fibromyalgia. Suicidal ideation was present in every group, mostly in patients with cluster headache and comorbid fibromyalgia. Suicidal ideation was severe in most of the patients: 29 (32%) patients in the total sample, 16 (41%) patients with cluster headache, 5 (33%) patients with cluster headache and migraine, 9 (90%) patients with cluster headache and fibromyalgia, and 10 (37%) patients with cluster headache, migraine and fibromyalgia reported having suicidal thoughts more than half the days or nearly every day. High ISI scores were noted across all subgroups. Eighty (87.9%) patients in the total sample had ISI scores above the cutoff point for insomnia. At the subgroup level, ISI scores exceeding the cutoff point were observed in 30 (76.9%) patients with noncomorbid cluster headache, 14 (93.3%) patients with cluster headache and comorbid migraine, 9 (90%) patients with cluster headache and fibromyalgia, and 27 (100%) patients with cluster headache and comorbid migraine and fibromyalgia. Both the EQ-5D-5L and EQ-5D-5L VAS scores were well below the mean values reported for the Spanish-speaking population, with the lowest scores observed in patients with cluster headache and comorbid migraine and fibromyalgia.

**Table 2.** Depression, suicidal ideation, sleep quality and health-related quality of life of the sample.

	Total sample (N = 91)	CH (N = 39)	CH + M (N = 15)	CH + FM (N = 10)	CH + M + FM (N = 27)	*p*	*p*-value* (CH *vs.* CH + M)	*p*-value* (CH *vs.* CH + FM)	*p*-value* (CH *vs.* CH + M + FM)
PHQ-9 scores mean ± SD and range	15.3 ± 5.8 (0–27)	14.8 ± 6.2 (0–28)	14.5 ± 7.0 (1–27)	13.9 ± 5.7 (2–21)	16.8 ± 4.6 (8–26)	0.530	0.999	0.999	0.754
Positive suicidal ideation N (%)	62 (68.1)	27 (69.2)	8 (53.3)	9 (90.0)	18 (66.7)	0.288	NA	NA	NA
ISI scores mean ± SD and range	18.0 ± 6.2 (1–28)	16.0 ± 6.7 (1–26)	18.1 ± 5.5 (10–27)	18.7 ± 6.8 (6–28)	20.9 ± 4.5 (12–28)	0.017	0.457	0.457	0.005
EQ-5D-5L scores mean ± SD and range	0.7360 ± 0.114 (0.514–0.799)	0.7822 ± 0.103 (0.582–1.000)	0.7951 ± 0.059 (0.694–0.010)	0.7153 ± 0.120 (0.533–0.910)	0.6450 ± 0.093 (0.514–0.857)	<0.0001	0.874	0.345	<0.0001
EQ-5D-5L VAS scores mean ± SD and range	47.1 ± 26.1 (0–90)	52.7 ± 24.9 (0–90)	53.7 ± 24.1 (10–90)	45.5 ± 29.0 (10–85)	35.9 ± 25.7 (0–90)	0.640	0.999	0.999	0.037

CH: cluster headache; FM: fibromyalgia; M: migraine; NA: not applicable; PHQ-9: Patients Health Questionnaire-9; ISI: Insomnia Severity Index; EQ-5D-5L: The 5-level EQ-5D version; VAS: visual analog scale; SD: standard deviation. *Post-hoc statistical testing was evaluated across subgroups for continuous variables.

Table 3 shows the HCRU data for the overall sample and the different subgroups. More than half of the patients in each group had visited a family physician or some specialist within the last six months. Regardless of the study subgroup, more than two-thirds of the patients had visited a family physician in the last six months, and a similar proportion of patients had also visited a specialist. The proportion of patients visiting the emergency room was also high (*i.e.*, more than 40%) in all subgroups, with the highest proportion among patients with all three comorbidities. Over 20% of the patients required hospitalization in the last 6 months. Finally, the need to undergo surgical intervention was also high; overall, 41 (45%) patients required surgical intervention.

**Table 3.** Use of health care resources in the last six months.

	Total sample	CH (N = 39)	CH + M (N = 15)	CH + FM (N = 10)	CH + M + FM (N = 27)	*p*
Family physician visits	67 (73.6)	25 (64.1)	10 (66.7)	8 (80.0)	24 (88.9)	0.130*
Specialist visits	69 (75.8)	31 (79.5)	9 (60.0)	8 (80.0)	21 (77.8)	0.478*
Emergency room visits	49 (53.8)	17 (43.6)	7 (46.7)	6 (60.0)	19 (70.4)	0.166
Hospitalization (>1 day)	24 (26.4)	11 (28.2)	3 (20.0)	2 (20.0)	8 (29.6)	0.864*
Surgical interventions	41 (45.1)	16 (41.0)	3 (20.0)	5 (50.0)	17 (63.0)	0.054
Clinical analyses	63 (69.2)	24 (61.5)	7 (46.7)	8 (80.0)	22 (81.5)	0.017*
Any	83 (91.2)	35 (89.7)	13 (86.7)	10 (100.0)	25 (96.2)	0.678*

CH: cluster headache; FM: fibromyalgia; M: migraine. *Chi-square calculations are only valid when all expected values are greater than 1 and at least 20% of the expected values are greater than five; as these conditions were not fulfilled, p values could not be valid.

## 4. Discussion

In our sample, 57.1% of the patients with cluster headache experienced comorbid fibromyalgia and/or migraine. The comorbidity with fibromyalgia was especially associated with an increased disease burden in terms of additional medical comorbidities, sleep disturbance, mood impairment, quality of life impairment, and heavy HCRU. Cluster headache, with or without associated pathologies, was also associated with a high frequency of suicidal ideation.

Patients who experienced cluster headache associated with fibromyalgia were predominantly female, presented a greater number of comorbid medical conditions, and had the highest depression and sleep disturbance scores and the lowest health-related quality of life scores. Furthermore, these patients utilized health care facilities more heavily than patients without comorbidities did. The prevalence of comorbid fibromyalgia among patients with cluster headache in our sample (40.6%) was strikingly greater than that found in previous studies: 6.7% in the sample of Joshi *et al*. [7], and 3% in the sample of Lund *et al.* [8]. This variability could be attributed mainly to differences among the study samples. In the study by Joshi *et al*. [7], patient data were obtained from a large health care registry, and in the study by Lund *et al*. [8], patient data were obtained from a survey of cluster headache patients who were visiting a tertiary headache clinic. It is also worth noting that the latter study, which found the lowest prevalence of comorbid fibromyalgia in patients with cluster headache, was not specifically designed to investigate cluster headache comorbidities. Instead, the study focused on cluster headache lifestyle patterns. On the other hand, the patients included in our study were headache association members. These patients are somewhat more concerned about their condition and more likely to seek support with respect to diagnostic and treatment options than the general population of patients with cluster headache [30]. Additionally, it is possible that patients with more severe disease and/or more associated comorbidities are more likely to become members of a patient association.

More than 60% of patients in each subgroup had clinically relevant depression scores, with the highest percentage in the subgroup of patients with cluster headache and comorbid migraine and fibromyalgia. As stated in the Introduction section, the association between depression and each of these three conditions has been well established [9, 10, 11]. Thus, it seems logical that the comorbidity of these conditions would likely exert a synergistic effect and increase the odds of depression. In fact, a recent study found that patients with cluster headache and comorbid migraine had the highest risk of moderate to severe depression and anxiety compared to both a control group without headache and a group of patients with only cluster headache [31].

The prevalence of suicidal ideation was also high in each subgroup, with the highest proportion observed in the subgroup of patients with comorbid cluster headache and fibromyalgia. Again, each of the three diseases has been associated with suicidal ideation, and cluster headache has even been referred to as “suicide headache” [12, 13, 14]. The prevalence of suicidal ideation with cluster headache was found to be 55% in U.S. patients. More recently, Ji Lee *et al.* [14] (2019) found that this prevalence was dependent of the headache phase, with both passive and active suicidal ideation highest during the ictal periods (64.2%) and substantially lower during the interictal periods (35.8%) [14]. In our study, the prevalence of suicidal ideation in patients with non-comorbid cluster headache was 69%; this higher rate is probably due to the fact that patients appertaining to a patients’ association are likely among those more severely affected by the disease. The prevalence of suicidal ideation among patients with migraine is substantially lower than in cluster headache, with a mean prevalence of 15.5% of the population, as shown in a recent meta-analysis [12]. In contrast, the rate of suicidal ideation in patients with fibromyalgia has been shown to range between 26 and 54% of the studied samples; thus, the 90% rate of suicidal ideation found in our study in patients with cluster headache and comorbid fibromyalgia does not seem disproportionate [32].

In addition to attacks being frequently triggered during nocturnal sleep, cluster headache has been linked to poor sleep quality [19, 33]. Several studies have shown that the Pittsburgh Sleep Quality Index scores are worse in the following patient populations: (a) patients with chronic cluster headache compared to those with episodic cluster headache [19, 33]; (b) patients with cluster headache with attacks triggered by sleep compared to those whose attacks were not sleep-triggered [19]; (c) during cluster attacks periods compared to headache-free periods [33]; (d) women than in men [34]. In patients with migraine, poor sleep quality is also more common in patients with chronic migraine than in patients with episodic migraine [35], and poor sleep quality has been identified as a stronger risk factor for migraine in women than in men [36]. Disturbed sleep is also prevalent in patients with fibromyalgia [18], with women exhibiting a greater incidence of sleep disturbance than men. The observed sex differences are in accordance with our findings, where the highest sleep disturbance scores were found in the subgroup of patients with cluster headache associated with migraine and fibromyalgia, followed by those with cluster headache and migraine and those with cluster headache and fibromyalgia; all of these groups had a female preponderance. According to the study by Calandre *et al*. [37] (2022), patients with fibromyalgia associated with migraine had a lower ISI score (18.4 ± 5.5) than did patients with cluster headache associated with migraine and fibromyalgia in our study. This finding suggests the presence of a potentially additive negative effect of each condition on sleep quality.

Our study showed that patients with cluster headache with or without comorbidities have poorer quality of life, as shown by the lower EQ-5D scores compared with those of the general Spanish-speaking population, even when compared with the data of people with associated medical conditions, which had a score of 0.864 for the EQ-5D-5L index and 71.42 for the EQ-5D-5L VAS. These findings are consistent with previous studies that documented the detrimental impact of cluster headache on patient quality of life; this impact was lower in patients with chronic cluster headache than in those with episodic cluster headache and was linked to a significant socioeconomic burden [38, 39]. Fibromyalgia and, to a lesser extent, migraine have also been linked to poor health-related quality of life [40, 41]. In patients with migraine, the EQ-5D-5L index score and the EQ-5D-5L VAS score were lower in patients with the chronic subtype than in those with the episodic subtype. The comorbidity of migraine and fibromyalgia has been shown to significantly decrease scores on all eight dimensions of the 36-Item Short Form Health Survey [42]. Thus, it is not surprising that, in our sample, patients with cluster headache with comorbid fibromyalgia and migraine had the lowest EQ-5D-5L index and ED-5D-5L VAS scores.

Finally, in line with the findings of previous studies [43, 44], our results indicate that CH is associated with heavy use of health care resources. Additionally, our data suggest that comorbidity with fibromyalgia, with or without migraine, could increase this burden; although the present study did not include a noncluster headache group, in a previous publication comparing patients with migraine, fibromyalgia and migraine with comorbid fibromyalgia, we found that patients with either fibromyalgia or migraine associated with fibromyalgia had greater HCRU than patients with migraine only [37]. This increase is especially reflected by a higher frequency of family physician visits, emergency room visits, surgical interventions and clinical analyses. This is likely associated not only with comorbidities associated with these clinical entities (*i.e.*, fibromyalgia and migraine) but also with other medical comorbidities that are more frequent in patients with CH and fibromyalgia. It would have been interesting to assess the causes of hospitalization and, especially due to the observed differences between groups, of surgical interventions; unfortunately, most patients did not answer the open-ended questions included in the survey related with this subject. Choong *et al.* [43] analyzed HCRU and costs among patients with CH in the U.S. and reported that CH was infrequently mentioned as the reason for increased HCRU. Thus, the authors concluded that comorbid conditions are the main drivers of the increased economic burden among these groups of patients [43].

The main strength of our study is that we specifically investigated the comorbidity of cluster headache with fibromyalgia, which has been largely disregarded in previous studies. This study, however, has several limitations. First, we enrolled patients who were members of a patient association that, as stated above, cannot be considered representative of the general cluster headache population. Nevertheless, this approach enabled us to enroll a relatively high number of patients diagnosed with this rare headache condition. Second, the fact that our study was based on an internet survey increases the possibility of recall bias in some of the responses, as well as the impossibility of controlling the reliability and validity of the collected data, as all of the questionnaires were self-reported. Third, our study did not distinguish between patients with episodic and chronic cluster headache or between episodic and chronic migraine patients. Fourth, in relation to HCRU, we did not obtain information on the specific reasons for hospitalization or the types of surgical interventions. Fifth, given the low prevalence of cluster headache, no specific sample size calculations were carried out because the objective was to include as many patients as possible. Finally, the sample size was limited, which requires careful interpretation of our findings.

## 5. Conclusions

We believe that our study emphasizes the need for further studies investigating the frequency and potential clinical relevance of the comorbidity of cluster headache and fibromyalgia, with or without concomitant migraine. We suggest that the risk of suicide in these populations should be further evaluated using a population-based study that increases representativeness, allows a better categorization of diagnoses, and includes a harder outcome measure such suicidal attempts and/or mortality due to suicide. Meanwhile, the potentially high rate of suicidal ideation in these patients suggests that careful screening and monitoring of the risk of suicide are of particular importance in clinical practice.

## 6. Clinical implications and highlights

• The comorbidity of cluster headache with fibromyalgia and/or with migraine seems to occur frequently.

• These comorbidities, especially those that involve fibromyalgia, increase the psychological burden of cluster headache and markedly decrease health-related quality of life.

• In these patients, particular attention should be given to the presence of suicidal ideation.
